# GCNet: A Deep Learning Framework for Enhanced Grape Cluster Segmentation and Yield Estimation Incorporating Occluded Grape Detection with a Correction Factor for Indoor Experimentation

**DOI:** 10.3390/jimaging11020034

**Published:** 2025-01-24

**Authors:** Rubi Quiñones, Syeda Mariah Banu, Eren Gultepe

**Affiliations:** Computer Science, Southern Illinois University Edwardsville, Edwardsville, IL 62026, USA; mariaahbanu@gmail.com (S.M.B.); egultep@siue.edu (E.G.)

**Keywords:** deep learning, grape segmentation, unsupervised learning, grape object detection, feature extraction, convolutional neural networks

## Abstract

Object segmentation algorithms have heavily relied on deep learning techniques to estimate the count of grapes which is a strong indicator for the yield success of grapes. The issue with using object segmentation algorithms for grape analytics is that they are limited to counting only the visible grapes, thus omitting hidden grapes, which affect the true estimate of grape yield. Many grapes are occluded because of either the compactness of the grape bunch cluster or due to canopy interference. This introduces the need for models to be able to estimate the unseen berries to give a more accurate estimate of the grape yield by improving grape cluster segmentation. We propose the Grape Counting Network (GCNet), a novel framework for grape cluster segmentation, integrating deep learning techniques with correction factors to address challenges in indoor yield estimation. GCNet incorporates occlusion adjustments, enhancing segmentation accuracy even under conditions of foliage and cluster compactness, and setting new standards in agricultural indoor imaging analysis. This approach improves yield estimation accuracy, achieving a R² of 0.96 and reducing mean absolute error (MAE) by 10% compared to previous methods. We also propose a new dataset called GrapeSet which contains visible imagery of grape clusters imaged indoors, along with their ground truth mask, total grape count, and weight in grams. The proposed framework aims to encourage future research in determining which features of grapes can be leveraged to estimate the correct grape yield count, equip grape harvesters with the knowledge of early yield estimation, and produce accurate results in object segmentation algorithms for grape analytics.

## 1. Introduction

Grapes (Vitis vinifera) are an economically significant crop grown worldwide, contributing to the wine, raisin, and fresh fruit industries. However, accurate yield estimation remains challenging, particularly due to occlusion in dense grape clusters [1]. Today, grapes are grown on more than seven million hectares of farmland across six continents, including key producers, Italy, the United States, and Australia [2,3]. Grapes are economically significant, supporting industries such as wine, raisins, grapeseed oil, and fresh fruit [1,2]. However, sustainable grape production faces challenges, particularly the need for precise vineyard monitoring to optimize crop development, which has led to the rise of precision viticulture [4]. Accurate yield estimation is crucial for guiding management practices—such as thinning, irrigation, and fertilization—while also improving logistical planning for storage and transportation [3,4,5]. Traditional yield estimation methods, which involve destructive sampling and manual counting, are labor-intensive and prone to errors, with discrepancies of up to 30% between estimated and actual yields [6,7]. These inaccuracies arise from the subjective nature of manual sampling and its inability to capture the variability across vineyards [2,7]. Additionally, the process is time-consuming and costly due to high labor demands [2]. This creates an urgent need for more accurate, non-invasive, and cost-effective alternatives for grape yield estimation [2].

Recent advances in imaging technologies and computer vision have offered promising solutions to the challenges of accurate yield estimation [8,9,10,11]. Object segmentation frameworks, widely used in agricultural settings, leverage deep learning techniques to detect and segment crops such as apples, oranges, and grapes, facilitating yield estimation in complex environments [1,12,13,14,15]. For example, Chen et al. [9] demonstrated the efficacy of convolutional neural networks (CNNs) in counting apples and oranges in orchards, achieving higher accuracy and faster processing than traditional manual methods [16]. Similarly, Xiao et al. [10] reviewed both traditional machine learning and modern deep learning approaches for detecting fruits and vegetables, emphasizing the growing role of deep learning in automating complex object recognition tasks in agriculture [17]. While these techniques have laid the groundwork for more sophisticated applications in fruit yield estimation, grape clusters persist with its own unique challenges compared to other fruits. Unlike apples or oranges, which are typically easy to detect individually, grape bunches often suffer from occlusion, with berries hidden by overlapping clusters or covered by canopy foliage. This occlusion, along with the compactness of grape clusters, complicates the task of accurately identifying all berries. Additional factors such as variable lighting conditions, dense foliage, and grape color variations can cause berries to blend into the background, further hindering accurate yield estimation [8,11]. While Palacios et al. [8] developed a deep learning framework for counting visible grape berries, their approach, like many others, did not fully account for occluded berries, leading to underestimation of the yield. Similarly, Santos et al. [18] combined deep neural networks with 3D association for grape detection and tracking, but this method still focused primarily on visible clusters. Although these frameworks have significantly advanced grape detection, their reliance on detecting only visible grapes limits their effectiveness for yield estimation. In contrast to fruits in more open environments, grape yield estimation requires models that can account for both visible and occluded berries to provide a more accurate assessment. This ongoing challenge underscores the need for novel solutions capable of addressing occlusion and improving the accuracy of grape yield predictions.

To address these challenges, we propose a novel deep learning framework called the Grape Counting Network (GCNet), which enhances segmentation accuracy by using a correction factor to account for occluded grapes, providing more accurate yield estimates. Additionally, we introduce a new dataset, GrapeSet, which includes Red–Green–Blue (RGB) imagery of grape clusters along with relevant supplemental such as grape count and weight. This dataset is designed to handle a wide range of grape colors and challenging imaging conditions, such as heavy foliage and obstructions, which are often met in real-world vineyard environments.

The key contributions of this paper are as follows:

1. Grape Counting Network (GCNet): A novel deep learning framework designed to address the issue of occluded berry counting using object segmentation and a correction factor.
2. GrapeSet: A new dataset for indoor experimentation having annotated images of grape clusters, including grape count and weight data, to enable more accurate yield estimation.
3. Ablation study: An evaluation of the role of segmentation in grape yield estimation, showing its necessity and impact on accuracy.
4. Efficacy study: Analysis of how image resolution, foliage density, and grape color affect the performance of GCNet in estimating grape yield.

## 2. Related Works

This section reviews the existing literature and datasets, highlighting key research gaps relevant to grape yield estimation. It introduces computer vision applications in agriculture, discusses feature-based and learning-based segmentation methods, and explains how our proposed network addresses their limitations. Finally, the section compares existing datasets, outlines their shortcomings, and presents the features of our new dataset designed to overcome these challenges.

### 2.1. Feature-Based Segmentation Methods

Fruit yield estimation is crucial for growers to manage their orchards and optimize harvest logistics [12]. Traditionally, machine learning algorithms have been used to estimate yields by exploiting features such as color [19,20], geometry [21,22], texture [23,24], and spatial arrangement of fruits [12]. Commonly used algorithms include decision trees [10,25,26], clustering techniques [27,28,29,30], and support vector machines (SVMs) [31]. For instance, Tanco et al. [30] evaluated the performance decision trees, SVMs with a Gaussian radial basis kernel [32], and K-Nearest Neighbor (KNN) for apple segmentation, achieving an F1 score of 0.91 with the KNN algorithm. Their study demonstrated the effectiveness of classification techniques in specific scenarios, but the model’s success was highly dependent on the uniformity of the apples in terms of color and shape. Any significant deviations from the dataset’s characteristics, such as variations in lighting or fruit orientation, could substantially reduce performance, highlighting the model’s lack of robustness to variability in orchard conditions. Similarly, Liu & Whitty [33] applied an SVM classifier for grape segmentation, leveraging color properties to reach an accuracy of 91.77%. While this result is promising, the model was trained specifically on purple grapes under controlled conditions. As acknowledged by the authors, the algorithm struggled to generalize to other grape varieties, such as green grapes, or under different environmental factors such as fluctuating lighting or heavy canopy coverage. This limitation underscores a critical challenge in traditional machine learning methods: their reliance on handcrafted features, such as color and texture, which often fail to adapt to new or unseen data. This lack of generalization restricts the adaptability of these algorithms to new datasets or environments [9] and likely require a learning-based approach.

### 2.2. Learning-Based Segmentation Methods

Deep learning algorithms have gained recognition for their superior ability to generalize across varying conditions while maintaining high levels of accuracy. Notably, these algorithms exhibit greater usability when applied to datasets containing multiple object types [18,31]. In 2017, Chen et al. [9] applied a deep learning pipeline to count both apples and oranges, achieving a mean squared error (MSE) of 13.8 for oranges and 10.5 for apples. Their segmentation accuracy, measured by the mean Intersection over Union (IoU), reached 0.838 for apples and 0.813 for oranges. This demonstrates the robustness of the same algorithm in handling two distinct fruits with differing colors, shapes, and textures, highlighting deep learning’s ability to generalize across different fruit types. Other studies [32,33] have also created a multi-fruit system for citrus fruits with accuracy of at most 97%.

For grape segmentation, Santos et al. [11] explored the capabilities of convolutional neural networks (CNNs) for segmenting grape clusters of various sizes. By comparing multiple CNN architectures, they found that Mask Region-based Convolutional Neural Network (R-CNN) achieved the highest performance, with an F1 score of 0.89. Other works that attempt grape segmentation [34,35] have achieved accuracies and Intersection-over-Union (IoU) of at most 88%. Compared to studies analyzing citrus fruits [36], these results indicate lower accuracies and Jaccard indices for grape segmentation. The authors attribute this discrepancy to the inherent complexity of grape structures, such as dense clustering of berries, heavy foliage, and color similarities among grapes, which make segmentation more challenging. Although recent advances in deep learning have enhanced the accuracy of grape segmentation, a persistent challenge is the ability to detect occluded grapes. Occlusion from foliage or overlapping clusters can result in substantial yield underestimations. This is particularly evident in grape clusters, where only a small fraction of the total berries is visible in typical images. Addressing this issue requires more specialized frameworks that can account for the unseen portions of the yield.

### 2.3. Occlusion in Cluster Segmentation

Occlusion is a significant challenge in grape cluster segmentation, where overlapping berries and dense foliage complicate accurate yield estimation and cluster detection in vineyard environments. Addressing this requires models capable of handling intra-cluster occlusion, where individual grape visibility is limited and background interference is high [37,38]. To overcome these challenges, researchers have employed methods such as multisource information fusion, attention mechanisms, and optimized network architectures. Each approach contributes unique strengths, yet limitations remain in fully resolving occlusion-specific issues in grape cluster segmentation.

A prominent strategy involves multisource information fusion, which combines (but not limited to) depth, color, and spatial data to improve cluster visibility in complex vineyard environments. For instance, Peng et al.’s [39] MultiFuseYOLO model, integrates grape and leaf data to enhance varietal recognition under heavy occlusion conditions, using the SynthDiscrim algorithm to improve precision across similar grape varieties. Similarly, Luo et al. [40] uses PointResNet, a model that achieved up to 96.5% segmentation accuracy by incorporating RGB and depth information within a 3D point cloud framework to better distinguish between overlapping grapes, foliage, and background structures in vineyard settings. Liang & Wang [41] designed a deep learning model for a picking robot to detect and harvest grapes. Their recognition achieved at most 80% accuracy. These models have shown to enhance segmentation accuracy, although computationally demanding for processing high-dimensional data. Koirala et al. [12] used a correction factor for mangoes, reducing error rates to 1.6% by accounting for occluded fruits. This method illustrates the potential of occlusion correction but would need adaptations for the denser, more structured layouts of grape clusters.

Attention mechanisms represent another prevalent technique in enhancing segmentation performance within challenging backgrounds. Models such as WineYOLO-RAFusion [39], which integrates a CFP-centered feature pyramid with a Res-Attention module, refine spatial feature extraction and segmentation in moderately occluded clusters. This method aligns with Häni et al.’s [13], U-Net model in apple segmentation, which achieves 97.83% accuracy by focusing on essential features. Huang et al. [42] further optimized Mask R-CNN by adding dual attention (DANet) and efficient channel attention modules, achieving a mask_mAP of 82.1% and a bbox_mAP of 90.5% for grape cluster segmentation in orchards. However, these models typically struggle with real-time application due to computational demands, which limit scalability in field-based applications. Yi et al.’s [43] applied dual-attention U-Net to grape disease segmentation, reaching pixel accuracy of 94.33% and a mean intersection-over-union (MIoU) of 91.09%, though similar methods require adjustments to segment tightly packed grapes in clusters.

To address processing efficiency, optimized network structures are employed to balance computational demands with segmentation accuracy. Wang et al.’s [44] knowledge distillation technique, for instance, effectively distills a large model into a compact student model, suitable for edge-device deployment. However, while this method retains accuracy in grape detection, it struggles with precision at the individual berry level within occluded clusters. Similarly, YOLOv8n-GP, developed by Jiang et al. [45], combines SENetV2 attention with CARAFE upsampling, reaches a precision of 91.6%, recall of 91.3%, and mAP of 97.1% for stem detection, although it lacks the granularity for dense intra-cluster segmentation. Meanwhile, Slaviček et al. [46] demonstrate that semi-supervised learning can reduce manual annotation requirements by up to 99%, producing extensive datasets with limited human input. This approach could be valuable for efficient dataset generation in vineyard settings where annotated data are limited.

While these techniques provide a strong foundation, studies such as Mohimont et al. [47] indicate an ongoing gap, noting that only 19% of grapes within a cluster are typically visible, which highlights the need for enhanced occlusion adjustment techniques. Additionally, Zabawa et al. [6] report frequent under-segmentation in dense grape clusters, pointing to the difficulties in achieving accurate yield estimates without advanced occlusion handling. While current segmentation advancements leverage multisource fusion, attention mechanisms, and optimized architectures to improve segmentation under occlusion, most models lack the specificity required for handling the unique occlusion patterns found within grape clusters. Furthermore, the complex nature of outdoor experimentation can lead to accuracy issues that may be hard to address or untangle for proper experimentation. Indoor experiments have attempted to detangle the outdoor complexity to propose frameworks for grape detection [41], segmentation [48], and yield estimation [49]. This gap highlights an opportunity for future research to develop lightweight, grape-specific models capable of precise segmentation and real-time field deployment, directly enhancing yield estimation accuracy and facilitating scalable automation within viticulture. Indoor experiments have attempted to untangle the outdoor complexity to propose frameworks for grape detection [41], segmentation [48], and yield estimation [49]. This gap highlights an opportunity for future research to develop lightweight, grape-specific models capable of precise segmentation and real-time field deployment, directly enhancing yield estimation accuracy and facilitating scalable automation within viticulture.

We propose a novel framework called the Grape Counting Network (GCNet) to address the limitations of existing approaches in berry occlusion and provide an additional method for indoor experimentation. GCNet tackles the challenge of occluded berries by integrating segmentation with a correction factor to estimate hidden grapes accurately. Using a CNN with a U-Net architecture, GCNet first segments the grape clusters and then applies a regression model that incorporates weight and count features to predict the true yield. By leveraging both visible and occluded grapes, GCNet provides a more accurate yield estimation, offering a significant improvement over previous methods that focus solely on visible fruits. This multi-stage approach not only improves segmentation accuracy, but also ensures that occlusion is accounted for, making GCNet a robust solution for real-world vineyard conditions where hidden berries often lead to yield underestimation. With its ability to generalize across various grape cluster densities and foliage conditions, GCNet represents a significant advancement in grape yield estimation.

### 2.4. Existing Grape Datasets

Developing robust models for grape segmentation and yield estimation is heavily dependent on the availability and quality of training datasets. Existing datasets often suffer from significant limitations, including a lack of diversity in grape colors, inconsistent imaging environments, limited ground truth annotations, and inadequate representation of varying levels of foliage. These constraints hinder the generalizability and real-world applicability of computer vision algorithms for viticulture.

Most existing grape datasets focus on outdoor vineyard conditions, capturing images against natural backgrounds with varying lighting, shadows, and weather conditions. While this approach reflects real-world settings, these variations can introduce inconsistencies that degrade algorithm performance. For instance, datasets such as the Segmentation of Berries Dataset [50] rely on uncontrolled outdoor environments, making it difficult to standardize imaging conditions. While some datasets, like the Segmentation of Berries Dataset with white backdrops and artificial lighting, attempt to address these inconsistencies, they fail to account for the natural variability of foliage density and grape occlusion. Moreover, few outdoor datasets provide the necessary ground truth annotations for accurate yield estimation, limiting their utility for training and evaluating segmentation algorithms [45,46].

Table 1 provides an overview of the features present in open-source datasets used in outdoor grape segmentation and yield estimation research. The Grape CS-ML dataset [3], one of the earliest open-source datasets, has paved the way for several subsequent datasets aimed at improving computer vision models for grape analysis. While many of these datasets focus on green-colored grapes, few include blue and purple grapes, despite the evidence presented by Liu & Whitty [33] that diverse grape colors improve the generalization of grape counting algorithms. Only two datasets, Grape CS-ML (versions 1–4) [3] and the Grapevine Bunch Detection Dataset [51], incorporate multiple colors.

**Figure 1:**
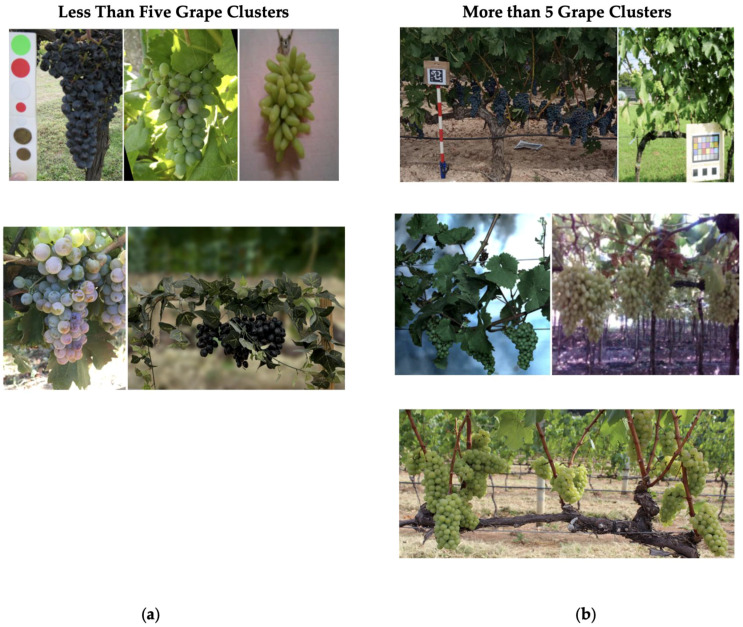
Images of grape clusters used in existing datasets, categorized by the number of grape clusters present in each image. (**a**) shows images with fewer than five grape clusters [37,38,42] including our GrapeSet, while (**b**) shows images containing more than five clusters [40,41,42].

In addition, the presence of varying levels of foliage in grape images is crucial for accurate yield estimation in real-world conditions [52]. As shown in Table 1, the existing datasets can be categorized based on the amount of foliage—Low, Medium, or High. For example, the Grapes CS-ML dataset has images with minimal occlusion and overlapping, classified as Low foliage. The Embrapa Wine Grape Instance Segmentation Dataset (WGISD) [53] represents Medium foliage, with some grape coverage by leaves. The wGrape UNIPD-DL dataset [54], on the other hand, features High foliage, where large portions of the grapes are obscured by leaves and overlapping clusters. Figure 1 demonstrates the impact of cluster quantity across various datasets. Another critical factor influencing dataset performance is the imaging environment. Most existing datasets, such as the Segmentation of Berries Dataset [50], feature images taken outdoors against natural green backgrounds. While this is realistic, variations in lighting, shadows, and environmental conditions can introduce inconsistencies that degrade algorithm performance [55,56]. The Segmentation of Berries Dataset addresses this by employing a controlled setup with a white backdrop and artificial lighting, ensuring consistent conditions across images. Lastly, accurate yield estimation relies on the availability of ground truth data. However, only two existing datasets provide ground truth annotations necessary for training segmentation algorithms.

While outdoor datasets are prevalent, there are no publicly available datasets (to the authors knowledge) for controlled indoor environments, despite the increasing research interest in indoor grape cultivation [57,58,59,60,61,62]. Indoor studies typically focus on optimizing photosynthesis, development, and grape quality but rarely integrate imagery for yield estimation. This represents a notable gap in viticulture research, as other agricultural fields, such as maize, sorghum, and sunflower, have successfully used imagery in indoor experiments to improve crop development [63,64,65,66,67,68]. These studies demonstrate how imagery can enhance resource efficiency, manipulate growth cycles, and improve crop quality, highlighting the untapped potential for similar advancements in indoor grape research.

**Table 1:** A summary of key features across various open-source grape yield estimation datasets for outdoor experimentation, including grape color, foliage level, background, acquisition settings, and the availability of supplemental data. X means that ground truth is not provided in the dataset, and ✓ means that ground truth is provided in the dataset.

Dataset	Grape Color	Foliage	Background	Acquisition	Num. of Images in Dataset	Num. of Grape Clusters in Each Image	Ground Truth	Supplemental Feature Data
Grape CS—ML Datasets (1–4) [3]	Blue, Green, and Purple	Low	Green Natural	Outdoor	2016	1	X	Volume and/or color references
Grape CS—ML Dataset (5) [3]	Green	Low	Green Natural	Outdoor	62	1–3	✓	Num. of berries, volume, pH, hue, bunch weight, TSS, etc.
Grapevine Bunch Detection Dataset [51]	Blue and Green	Low, Medium	Green Natural	Outdoor	910	1–3	X	Annotation and condition of grape bunch
Embrapa WGISD [53]	Green	Medium	Green Natural	Outdoor	300	5–25	X	Boxed and masked cluster annotations
AI4Agriculture Grape Dataset [67]	Blue	High	Green Natural	Outdoor	250	5–25	X	Annotations of bounding boxes
wGrapeUNIPD-DL [54]	Green	High	Green Natural	Outdoor	373	5–25	X	Color reference
GrapeNet Dataset (3) [68]	Green	Low	Black, Coral, and Green Natural	Outdoor	1705	1–2	X	Augmented images
Segmentation of Wine Berries [50]	Green	High	White	Outdoor	42	5–25	✓	Labels of berries
GrapeSet (ours)	Blue, Green, and Purple	Low, Medium, and High	White and Green Bokeh	Indoor	2160	3	✓	Weight of grape bunches, actual count of berries

In response to these challenges, we introduce GrapeSet, a novel dataset designed to address the limitations of existing resources. GrapeSet includes images of green, blue, and purple grapes, ensuring color diversity that improves model generalization. The dataset also features varying levesl of foliage—Low, Medium, and High—to simulate diverse occlusion scenarios. Unlike most datasets, GrapeSet was imaged indoors providing controlled conditions to eliminate variability caused by lighting and shadows. Additionally, GrapeSet includes detailed ground truth annotations, along with supplemental data such as grape count and weight, making it the most comprehensive dataset available for accurate yield estimation. These contributions position GrapeSet as a foundational tool for advancing grape segmentation and yield estimation, particularly in indoor environments.

## 3. Proposed Method

To overcome the challenges of cluster segmentation for grape yield estimation for indoor experimentation posed by occlusions and dense cluster formations, we propose GCNet, a deep learning framework. GCNet leverages a multi-stage approach to accurately identify both visible and hidden grapes, thereby improving yield estimation under complex vineyard conditions.

In the first stage, GCNet employs a U-Net-based segmentation model to isolate grape clusters from background elements and foliage. The segmented output is then refined in the second stage by overlaying a mask to remove background interference, enhancing focus on grape clusters alone. In the final stage, a correction factor is applied using a regression model to estimate the count of occluded grapes, ensuring a more accurate yield prediction. This section provides a detailed breakdown of each stage in GCNet, including its architectural components and the specific strategies used to enhance segmentation accuracy and yield estimation in occlusion-prone environments.

### 3.1. Problem Definition

Let $D=\left\{I_{1},I_{2},\ldots I_{n}\right\}$ represent a dataset of images, where each $I_{i}\in D$ contains one mor more grape clusters. The goal is to accurately estimate the total grape count $C_{i}$ for each image $I_{i}$ where $C_{i}$ includes both visible and occluded grapes. In this context, we face two primary challenges: (1) occlusion of grapes within clusters due to dense foliage or overlapping berries, (2) background interference from surrounding vineyard elements, which complicates accurate segmentation.

To formalize, let:

- $V_{i}\subset I_{i}$ denote the set of visible grapes in image $I_{i}$
- $O_{i}\subset I_{i}$ denote the set of occluded (hidden) grapes in image $I_{i}$.

The true grape count for image $I_{i}$ is then defined as:

$$C_{i}=\left|V_{i}\right|+|O_{i}|$$

where $\left|V_{i}\right|$ and $\left|O_{i}\right|$ represent the cardinalities of the visible and occluded grape sets, respectively. The task of GCNet is to predict ${\hat{C}}_{i}$, an estimate of the true count $C_{I}$, by approximating both $\left|V_{i}\right|$ and $\left|O_{i}\right|$ from $I_{i}$. We define this estimation process as a mapping f:I→R, where $f\left(I_{i}\right)={\hat{C}}_{i}$, such that ${\hat{C}}_{i}\approx C_{i}$.

GCNet approaches this problem through a three-stage framework:

1. Segmentation of Grape Clusters: A function s:I→M segments the imge $I_{i}$ to produce a mask $M_{i}$ that identifies grape clusters, thereby isolating $V_{i}$ from background elements.
2. Mask Overlay: The segmented mask $M_{i}$ is refined to reduce background noise and highlight grape clusters.
3. Generating Final Count: A correction factor is applied via a regression model r:M→R that estimates $\left|O_{i}\right|$, adjusting ${\hat{C}}_{i}=\left|V_{i}\right|+r\left(M_{i}\right)$ to account for occluded grapes.

Through this multi-stage process, GCNet aims to minimize the error $\left|C_{i}-{\hat{C}}_{i}\right|$ across all images in D, providing an accurate and reliable yield estimate even under comple occlusion conditions. The proposed framework is detailed in Algorithm 1.
**Algorithm 1** Proposed Grape Counting Network (GCNet)1:**Input:** A dataset D of size s with images of grapes i, where 1≤i≤s.2:**Output**: The number of grapes C where $C_{i}$ is the generated count for the image i. **Stage 1: Segmentation of Grape Clusters**3:**begin**4:**for** i = 1 to s **do**5:   $S_{i}\leftarrow SEGMENT(D_{i})$
6:**end for**7:**return** S8:**end** **Stage 2: Overlaying**9:**begin**10:**for** i = 1 to s **do**11:   $O_{i}\leftarrow SEGMENT(S_{i})$
12:**end for**13:**return** O14:**end** **Stage 3: Generating Final Count**15:**begin**16:**for**  i = 1 to s **do**17:   $C_{i}\leftarrow COUNT(O_{i})$
18:**end for**19:**return** C20:**end**

### 3.2. Overview

GCNet is designed as a multi-stage framework to address the unique challenges of grape segmentation and yield estimation, where occlusions and background interference frequently complicate accurate segmentation. The framework operates in three key stages, each tailored to improve accuracy by isolating visible grape clusters and compensating for occluded grapes. In the first stage, GCNet employs a U-Net-based segmentation architecture to identify and mask grape clusters in each image, distinguishing them from background elements. The second stage involves refining this mask to enhance the focus on grape clusters while minimizing background noise. In the third stage, a correction factor is applied using a regression model, estimating the count of occluded grapes and adjusting the overall yield prediction accordingly using grape count and weight. This structured approach allows GCNet to overcome limitations in traditional segmentation methods and achieve more accurate segmentation and yield estimations.

#### 3.2.1. CNN-Based U-Net Model

The U-Net model [69] is a deep learning model that is underpinned by a CNN architecture, which is modified specifically for image segmentation tasks. A CNN [70] is a type of deep neural network that is based on the feed-forward propagation of error gradients in a neural network, but rather than using fully-connected hidden layers for learning the features, locally constrained filters/kernels with a fixed receptive field are used to sample the data. Additionally, pooling layers, which reduce the feature space are interspersed among the learned convolutional filters. Thus, the relevant features are learned without overfitting and allow for deeper networks (i.e., multiple hidden layers). The main innovation the U-Net model has in addition to the standard CNN architecture is that the hidden layers are organized in an encoder-decoder manner, using transpose convolutions in the decoder portion of the network. The segmentation of the input images are provided by the final two-channel output (i.e., mask vs. background).

#### 3.2.2. Stage 1: Segmentation of Grape Clusters

**Figure 2:**
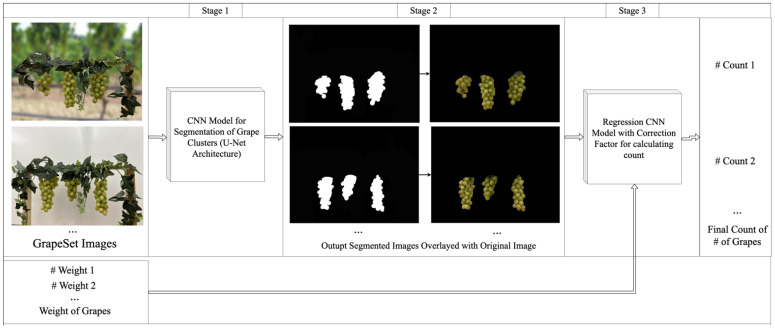
Our proposed GCNet framework.

A dataset of images of grapes are used as input to GCNet. First, in the segmentation step of the framework, the input images are passed through the U-Net model, as shown in Figure 2, to create a segmented mask that indicates where the grape clusters are located in an image. The U-Net model was tuned to with the following hyperparameters: Adam optimizer with a learning rate of 0.001, a cross-entropy loss function, 50 training epochs using, batch size of 8, and an 80–20 train–test split. Particular care was taken to ensure that no overlap occurred between the training and testing datasets. Although each grape bunch was captured in multiple images from different perspectives, the dataset was manually split to guarantee that all images of a specific grape bunch were entirely contained within either the training set or the testing set. This controlled splitting process ensured that images used for training were distinct from those used for evaluation, eliminating the possibility of data leakage and providing a fair evaluation of the model’s performance.

#### 3.2.3. Stage 2: Mask Overlay

The segmented mask outputted from Stage 1 is then overlayed with the original image to generate a simplified image in which only the grape clusters are shown, and the background pixels are omitted. This attempts to eliminate the confusion that can be caused by background pixels while calculating yield.

#### 3.2.4. Stage 3: Generating Final Count

Next, the simplified image is given as input to a regression CNN model to generate the final count of berries in each image of the dataset. This regression model uses a combination of convolutional and pooling layers which are flattened and followed by dense layers to generate the desired yield estimate. The CNN model was tuned to have the hyperparameters of an Adam optimizer with a learning rate of 0.001, which was trained for 50 epochs using batch sizes of 8 and an 80–20 train–test split. The regression model also takes weight and actual count as input to offset for the berries that are not visible in the image thus generating a truer yield estimate.

### 3.3. Implementation

The implementation of the proposed framework was carried out using Python (v3.10), leveraging several libraries to streamline data processing, model training, and evaluation. The TensorFlow/Keras library (v2.8) was employed to design and train a multi-output deep learning model for predicting grape count and weight from vineyard images. OpenCV (v4.5) was used for image preprocessing tasks, including resizing to a uniform size of 480 × 640 pixels and normalization to a 0–1 scale. Data organization and manipulation were handled using NumPy (v1.22) and pandas (v1.4), while Matplotlib (v3.5) was utilized for visualizing both the image data and the model’s performance metrics.

The pipeline began with data preprocessing to prepare the images and extract corresponding labels for supervised learning tasks. The model architecture featured convolutional layers for extracting spatial features, pooling layers for dominality reduction, and dense layers for the final predictions. Two separate output layers were included to independently predict grape count and weight. The training process employed the Adam optimizer with a learning rate of 0.001, optimizing the mean_squared_error loss for both outputs. Evaluation metrics included mean absolute error (MAE) and accuracy, calculated as the percentage deviation from the true values. MAE has been consistently used in previous studies such as Zabawa et at. [6], Sozzi et al. [4], and Santos et al. [11].

The framework was trained for 50 epochs, with all preprocessing steps, hyperparameters, and code thoroughly documented to ensure reproducibility. The complete implementation is available at https://github.com/rubiquinones/GCNet (accessed on 22 January 2024).

## 4. Proposed Dataset

This section introduces GrapeSet, a novel and publicly available dataset (https://doi.org/10.5281/zenodo.14019981), accessed on 22 January 2024. designed to evaluate the performance of computer vision algorithms in grape segmentation and yield estimation. GrapeSet was specifically developed to support the experimental validation of the proposed GCNet framework and is intended to address the limitations of existing datasets by offering a more comprehensive range of conditions, including multiple grape colors, varying levels of foliage, and detailed ground truth annotations.

### 4.1. Imaging Setup

The images in GrapeSet were captured in a controlled indoor environment to mitigate the variability in lighting and shadows commonly encountered in outdoor imaging. A white backdrop was employed to provide a neutral background, reducing distractions and ensuring that the focus remains on the grape clusters. The setup consisted of real grape clusters on a square wooden arch, complemented by artificial foliage, to simulate the natural environment while maintaining consistent conditions for image acquisition. An iPhone 13 Pro Max, equipped with a 12-megapixel camera, was used to capture the images at a resolution of 3024 × 4032 pixels. The decision to use a high-resolution camera was driven by the need for detailed imagery that can support precise segmentation and yield estimation algorithms. The camera was positioned on a tripod to maintain stability and consistency across images, while the grapes were manually rotated on a swivel to obtain multiple viewpoints. Specifically, each grape bunch was imaged at four angles—0, 90, 180, and 270 degrees—to provide diverse perspectives and simulate real-world scenarios where clusters may be viewed from various angles. By capturing multiple views of each grape bunch, GrapeSet ensures that occlusion, lighting variations, and cluster orientation are accounted for, making the dataset highly valuable for training models that need to generalize to different grape cluster configurations.

### 4.2. Dataset Organization

**Figure 3:**
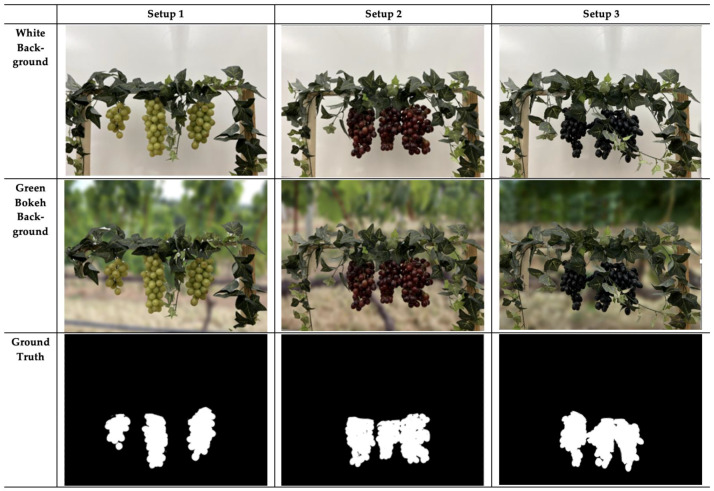
Sample images from GrapeSet of green, purple, and blue grapes captured in three different foliage setups: Setup 1 (Low Foliage), Setup 2 (Medium Foliage), and Setup 3 (High Foliage), respectively. Each row represents a different background—White (top row) and Green bokeh (middle row) backgrounds. The bottom row displays the corresponding ground truth masks for each setup, showing the precise location of the grape clusters.

To create a diverse dataset with many useful features, several variations were incorporated during the imaging process. GrapeSet includes images of three different grape color varieties: Blue, Green, and Purple. Additionally, as depicted in Figure 3, images were captured with three levels of foliage—Low, Medium, and High—to introduce varying segmentation challenges:

- Setup 1 (Low Foliage): Minimal foliage coverage, with grapes clearly visible and non-overlapping.
- Setup 2 (Medium Foliage): Grapes are closer together, overlapping slightly, and covered by more foliage, increasing the complexity of segmentation.
- Setup 3 (High Foliage): An additional layer of foliage is introduced, particularly with blue grapes, significantly increasing the difficulty of detecting and segmenting the clusters.

In addition to foliage variation, GrapeSet introduces different background settings to further challenge segmentation models. Alongside images taken against a plain white background, images were also captured with a simulated green bokeh background. The white background was the actual wall used during imaging, while the green bokeh background was designed to simulate the complexity of natural outdoor environments. This variation mimics the real-world challenges of outdoor imagery while maintaining controlled conditions for indoor imaging. Existing datasets typically feature either a green natural background or white/coral, but the green bokeh in GrapeSet introduces a new dimension.

GrapeSet also includes images captured at three different resolutions—High Resolution (1080 × 1440), Medium Resolution (480 × 640), and Low Resolution (120 × 160)—to explore the relationship between image quality and the accuracy of computer vision algorithms. By including multiple resolutions, GrapeSet allows researchers to investigate how varying image quality affects the performance of grape yield estimation models.

Each image in GrapeSet contains three grape clusters, with a total of 10 unique grape clusters (labeled A-J) used to create the dataset. These clusters were combined randomly into groups of three, ensuring that each cluster appeared approximately the same number of times in the dataset. For instance, an image labeled ‘A-B-C’ includes the grape clusters labeled A, B, and C. Detailed ground truth annotations were generated for each image using Adobe Photoshop 2023. These annotations consist of binary masks that indicate the precise location of the grape clusters within the image, serving as critical input data for training segmentation models.

## 5. Experiment Setup

The experimental framework for evaluating the performance of GCNet is divided into two major studies: the Ablation Study and the Segmentation Study. Each study includes experiments focused on image resolution and grape color to assess their influence on GCNet’s performance in grape yield estimation and segmentation accuracy.

### 5.1. Ablation Study

Ablation studies, originally derived from neuroscience, help researchers understand the significance of individual components within a system by selectively removing or altering parts [71]. In the context of computer vision, ablation studies allow for the simplification of models, making it easier to evaluate the contribution of specific elements. Aich & Stavness [72] conducted an ablation study to assess whether segmentation would improve leaf counting accuracy in Arabidopsis and Tobacco plants. Their findings showed that segmentation was beneficial for most experiments, except for some involving specific imagery of tobacco plants. For GCNet, the ablation study focuses on the role of segmentation in grape yield estimation. To evaluate its necessity, the experiment involves two phases: first, segmentation is removed from the framework, and the grape count is predicted directly from the original images. Then, segmentation is reintroduced, and the results are compared. This approach helps determine whether segmentation is critical for achieving high accuracy in yield estimation. The study is divided into two experiments:

#### 5.1.1. Resolution Experiment

In imaging, image resolution plays a crucial role in model accuracy. Previous studies, such as those by Zabawa et al. [6], used high-resolution images (2592 × 2048 pixels) and achieved an F1 score of 0.89. In contrast, Sozzi et al. [4] used resized low-resolution images for YOLOv4 and attained an F1 score of 0.77. To explore the influence of resolution on GCNet’s performance, this experiment involves three resolutions: Low (120 × 160), Medium (480 × 640), and High (1080 × 1440). The Mean Average Error (MAE) and R² metrics were used to assess the accuracy of grape yield estimation. MAE calculates the average magnitude of errors in predictions (Equation (1)), while R² evaluates the model’s overall fit (Equation (2)). These metrics are commonly used in the literature to measure the accuracy of regression models. In these equations, $y_{i}$ represents the actual observed values, and $\hat{y_{i}}$ denotes the predicted values generated by the model. For yield estimation, $y_{i}$ corresponds to the observed weight of grapes, measured in grams (g), while for cluster analysis, $y_{i}$ is the number of grapes in a cluster. Similarly, $\hat{y_{i}}$ represents the predicted weight in grams or the predicted grape count, matching the respective units of $y_{i}$.

Equation (1), which defines the MAE is expressed as:

(1) $$MAE=\frac{1}{n}\sum_{i=1}^{n}\left|y_{i}-{\hat{y}}_{i}\right|$$

where n is the total number of observations. MAE measures the average magnitude of errors between the predicted and observed values, providing an intuitive assessment of prediction accuracy. The unit of MAE is the same as the target variable—grams (g) for yield estimation and count for grape cluster analysis.

Equation (2), which defines the coefficient of determination (R^2^), evaluates the proportion of variance in $y_{i}$ explained by the predictions ($\hat{y_{i}}$). It is formulated as:

(2) $$R^{2}=1-\frac{SS_{residual}}{SS_{total}}$$

where $SS_{residual}$ is the sum of squares of the residuals, $\sum_{i=1}^{n}{\left(y_{i}-\hat{y_{i}}\right)}^{2}$, and $SS_{total}$ is the total sum of squares, $\sum_{i=1}^{n}{\left(y_{i}-\hat{y_{i}}\right)}^{2}$, with $\hat{y_{i}}$ representing the mean of the observed values. For yield estimation, the SS values are expressed in grams squared (g^2^), while for grape count analysis, they are in count squared. High R^2^ values indicate a strong correlation between the observed and predicted values, demonstrating the model’s ability to capture the variance in the data.

#### 5.1.2. Color Experiment

Understanding how grape color affects model performance is essential, as most previous datasets consist of a single grape color. In this experiment, images of blue, green, and purple grapes from the GrapeSet dataset were used to assess the impact of grape color on GCNet’s performance. Each color was tested separately using medium-resolution images across different backgrounds (white and green bokeh) and foliage levels (low, medium, and high). Similar to the Resolution Experiment, the MAE and R² metrics were used to evaluate performance. This experiment aimed to determine if different grape colors influence the model’s ability to estimate yield accurately and whether segmentation enhances performance for specific colors, particularly green grapes, which may blend with their foliage.

### 5.2. Segmentation Study

The segmentation study focuses on evaluating the performance of the U-Net CNN model used in GCNet for grape cluster segmentation. The performance is measured by the segmentation accuracy using two metrics: Intersection over Union (IoU) and F1 score. Two experiments were conducted in this study:

#### 5.2.1. Resolution Experiment

This experiment evaluates how image resolution affects the segmentation accuracy of the U-Net model. Similar to the Resolution Experiment in the ablation study, images from GrapeSet were resized into low, medium, and high resolutions. The model’s performance was measured using IoU and F1 score, which are widely accepted metrics in segmentation tasks.truth masks (Equation (3)). It ranges from 0 to 1, with 1 indicating perfect overlap. IoU measures the overlap between predicted and ground truth segmentation masks (Equation (3)), with values ranging from 0 to 1, where 1 indicates perfect overlap.

(3) $$IoU=\frac{Area\;of\;Overlap}{Area\;of\;Union}$$

The F1 score, useful for imbalanced datasets (e.g., where background pixels vastly outnumber grape pixels), evaluates the balance between precision and recall (Equation (4)).

(4) $$F1=2\times \frac{Precision\times Recall}{Precision+Recall}$$

Precision and recall are calculated as:

(5) $$Recall=\frac{True\;Positives}{True\;Positives+False\;Negatives}$$

(6) $$Precision=\frac{True\;Positives}{True\;Positives+False\;Positives}$$

This experiment was designed to ensure the segmentation model accurately identifies grape clusters at various resolutions, a critical factor for yield estimation. The metrics were leveraged from existing literature from Zabawa et al. [6] and Santos et al. [11].

#### 5.2.2. Color Experiment

This experiment explores how the color of grape clusters affects the segmentation model’s performance. Grape clusters of different colors pose different challenges for segmentation, particularly green grapes, which may be harder to distinguish from the green foliage and background. The IoU and F1 score metrics were again used to measure segmentation accuracy. This experiment aimed to identify how well the U-Net model could segment grape clusters of varying colors, and whether specific colors posed more significant challenges, especially in conditions of low contrast between the grapes and the surrounding environment.

## 6. Experimental Results

This section presents the results of the experiments conducted to evaluate the performance of GCNet. The results are organized based on our two major studies: the Ablation Study and the Segmentation Study.

### 6.1. Ablation Study

The ablation study was designed to assess the significance of the segmentation component in GCNet’s performance for grape yield estimation. By removing segmentation from the framework and comparing it to results with segmentation, we can determine how critical this step is for accurate predictions. Two experiments were conducted as part of this study: (1) Resolution Experiment: To evaluate how image resolution affects the model’s performance, both with and without segmentation, and (2) Color Experiment: To explore how grape color influences the model’s accuracy and the effect of segmentation in different color scenarios.

#### 6.1.1. Resolution Experiment

Table 2 summarizes the results of the ablation study performed across varying image resolutions. The study compares the performance of GCNet in estimating grape yield, both with and without the segmentation module.

Without segmentation, the model achieved an average R^2^ value of 0.88, indicating a fairly good fit. However, the inclusion of segmentation led to a noticeable improvement, raising the average R² to 0.90. The high R^2^ value (0.9) observed for yield estimation reflects the model’s ability to generalize across the dataset and is not the result of overlap between training and testing data. The manual split and careful cross-validation procedures reinforce the validity and reliability of the results. This improvement highlights the importance of segmentation for better yield estimation, especially in certain conditions. The most prominent enhancement in model performance was observed in low-resolution images.

When segmentation was introduced, the R^2^ value increased from 0.85 to 0.88, while the MAE dropped from 29 to 26. These improvements demonstrate that segmentation plays a critical role in enhancing the model’s ability to accurately estimate grape yield when working with lower-resolution images. Similarly, for medium and high-resolution images, smaller but consistent improvements were observed. The MAE for medium-resolution images improved by 1 point (from 22 to 21), and for high-resolution images, the MAE decreased from 20 to 19. This shows that, while the improvements in performance are more pronounced at lower resolutions, segmentation still offers a measurable benefit at higher resolutions.

These findings suggest that, after a certain resolution threshold is reached, the trade-off between increasing image resolution and performance improvements becomes smaller. Segmentation is particularly beneficial for lower-resolution images, where the finer details of the grapes are less discernible. This experiment demonstrates that the addition of segmentation is essential for improving model performance, especially when dealing with lower-resolution imagery.

#### 6.1.2. Color Experiment

The color experiment was conducted using medium-resolution images of three commonly found grape colors: blue, green, and purple. The results, shown in Table 3, demonstrate how segmentation influenced the model’s performance for each grape color. The most significant impact of segmentation was observed with green grapes. After incorporating segmentation into the framework, the R² value increased by 0.3 points, from 0.82 to 0.85, while the MAE dropped from 33 to 29. This improvement highlights the value of segmentation, particularly for green grapes, which are more challenging for the model due to their similarity in color to the surrounding foliage. While the performance improvements for blue and purple grapes were less pronounced, segmentation still contributed to a consistent enhancement in accuracy. For blue grapes, the R² improved slightly from 0.95 to 0.96, with no change in MAE. Similarly, for purple grapes, the R² remained at 0.91, with the MAE decreasing from 21 to 20.

These results suggest that segmentation is particularly beneficial for scenarios involving green grapes, where color similarity between the grapes and the foliage poses a challenge. In contrast, for blue and purple grapes, which offer higher contrast against the background, segmentation provides a smaller, but still measurable, improvement in model performance. Therefore, adding segmentation is crucial when dealing with color complexities, especially in challenging environments like those with green grapes.

### 6.2. Segmentation Study

The segmentation study focuses on evaluating the effectiveness of the U-Net CNN model used in GCNet for segmenting grape clusters. Accurate segmentation is critical for yield estimation, as it allows the model to isolate grape clusters from the background and other visual distractions. To assess the performance of the segmentation model, two experiments were conducted: (1) Resolution Experiment: To determine how different image resolutions affect the segmentation accuracy of GCNet, and (2) Color Experiment: To analyze the impact of grape color on the model’s segmentation performance, particularly in challenging environments such as green grapes blending with foliage.

#### 6.2.1. Resolution Experiment

**Figure 4:**
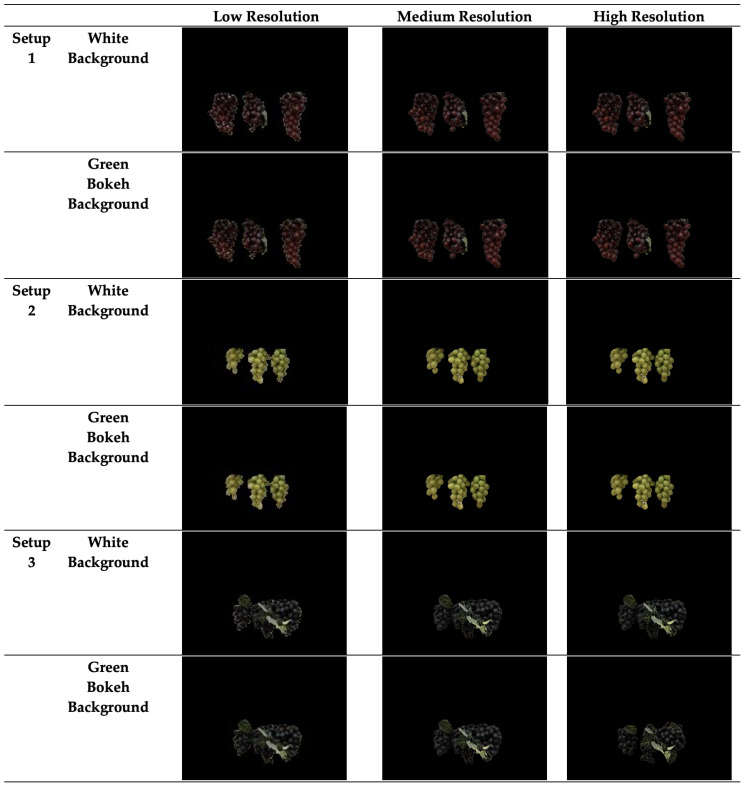
Qualitative segmentation results of grape clusters at varying resolutions (Low, Medium, High) across different setups and background types.

The segmentation model in GCNet was evaluated across varying image resolutions, and the results are presented in Figure 4 and Table 4. The model’s performance was measured using Intersection over Union (IoU) and F1 score, two common metrics used for assessing segmentation accuracy. The U-Net CNN model achieved an average IoU of 0.87 and an average F1 score of 0.95 across all resolutions, indicating strong segmentation capabilities overall.

The quantitative data in Table 4 clearly shows that the segmentation accuracy consistently improves as the image resolution increases. For low-resolution images, the model achieved an IoU of 0.83 and an F1 score of 0.93. These results suggest that while the model performs reasonably well at lower resolutions, a significant portion of the image detail is lost, making it harder for the model to correctly segment all parts of the grape clusters. This lower performance is expected due to the coarse granularity in the low-resolution images, which limits the amount of pixel information available to the model. As the resolution improves to medium, we observe a noticeable enhancement in the segmentation accuracy, with the IoU increasing to 0.87 and the F1 score improving to 0.95.

The medium-resolution images provide more detail, allowing the model to better distinguish between the grape clusters and the background. The increase in both IoU and F1 score indicates that the model is able to capture more accurate boundaries of the grape clusters at this resolution, leading to fewer segmentation errors.

Finally, for high-resolution images, the model achieves its best performance, with an IoU of 0.90 and an F1 score of 0.96. At this resolution, the model is able to fully utilize the detailed pixel information, accurately identifying the shape, edges, and boundaries of the grape clusters. The high IoU value indicates that the overlap between the predicted segmentation masks and the ground truth is nearly complete, while the F1 score shows that the model maintains a strong balance between precision and recall.

Overall, the quantitative results highlight the strong dependence of segmentation accuracy on image resolution. Higher-resolution images provide the model with more detailed information, resulting in better segmentation performance. This is especially important in tasks such as grape cluster segmentation, where small errors in boundary detection can significantly affect yield estimation accuracy.

The qualitative results of the resolution experiment (Figure 4) reveal notable differences in segmentation accuracy across low-, medium-, and high-resolution images, as observed in the three setups with varied backgrounds. In Setup 1 with a white background, the low-resolution images display considerable segmentation challenges. The model struggles to capture the intricate details of grape clusters, particularly the leftmost bunch, where portions of the clusters are either missing or inaccurately segmented. This under-segmentation is likely due to the coarse pixel structure, which limits the model’s ability to differentiate between the grape clusters and the surrounding background.

At medium resolution, there is a visible improvement in the segmentation output. The grape clusters appear more complete, and the model achieves a better outline of the clusters. However, minor inaccuracies persist, especially around the cluster edges, indicating that medium resolution provides more detail but is still insufficient for precise segmentation. High-resolution images yield the most accurate segmentation results in this setup, with the model producing nearly perfect segmentation masks that closely follow the contours of each grape cluster. The high pixel density enables the model to capture fine details, resulting in clear boundaries that match the actual shape of the grape clusters with minimal errors. In Setup 2, which introduces a green bokeh background, the segmentation model faces additional challenges, particularly at low and medium resolutions. The similarity in color between the green background and grape clusters causes the model to confuse parts of the background with grape clusters, leading to over-segmentation and occasional false positives.

At low resolution, this effect is particularly pronounced, with the model failing to distinguish clear boundaries due to the blending effect created by the green background. Medium resolution improves this somewhat, but residual noise along the edges of the grape clusters is still apparent. High resolution once again enhances the model’s performance, providing well-defined cluster boundaries even in the more complex green background environment.

The high-resolution segmentation masks demonstrate a clear distinction between the grape clusters and the background, with only minor edge imperfections. This outcome highlights the model’s reliance on higher-resolution data to accurately segment clusters when background interference is present. Finally, Setup 3 reinforces the observed trends by challenging the model with different orientations and varied foliage density.

As with the other setups, low-resolution images yield the least accurate segmentation, with several gaps in the clusters and incomplete boundaries that hinder precise yield estimation. Medium resolution offers moderate improvement, where the model begins to resolve more cluster details but still lacks full clarity. In contrast, high-resolution images allow the model to fully capture the clusters, even in complex foliage settings, leading to segmentation masks that are almost indistinguishable from the actual grape clusters. Across all setups, these qualitative results confirm that high-resolution images are essential for accurate segmentation, especially when complex backgrounds or low contrast with the background are present. This dependency on resolution is critical for applications in yield estimation, where precise cluster boundaries directly impact yield accuracy.

The results from both the quantitative and qualitative analyses demonstrate the significant impact that image resolution has on the performance of the segmentation model. As the resolution increases, the model is able to leverage more detailed pixel information, resulting in higher segmentation accuracy. For low-resolution images, the model still performs reasonably well, but its ability to capture fine details is limited, leading to incomplete or imprecise segmentation masks. This suggests that while the model can generalize across lower-quality images, its full potential is realized when provided with higher-resolution input. The improvement seen in medium and high-resolution images underscores the importance of using higher-quality images for tasks requiring precise segmentation. As the resolution increases, the model’s segmentation becomes more accurate, leading to better overlap between predicted masks and ground truth, as reflected in the improved IoU and F1 scores. This result is crucial for practical applications of GCNet in grape yield estimation, where accurate segmentation directly affects the final yield predictions. In conclusion, the Resolution Experiment shows that high-resolution images bring out the best performance from the segmentation model, achieving the highest accuracy with minimal segmentation errors. This suggests that in real-world applications, using higher-resolution images would significantly enhance the performance of grape yield estimation systems like GCNet.

#### 6.2.2. Color Experiment

**Figure 5:**
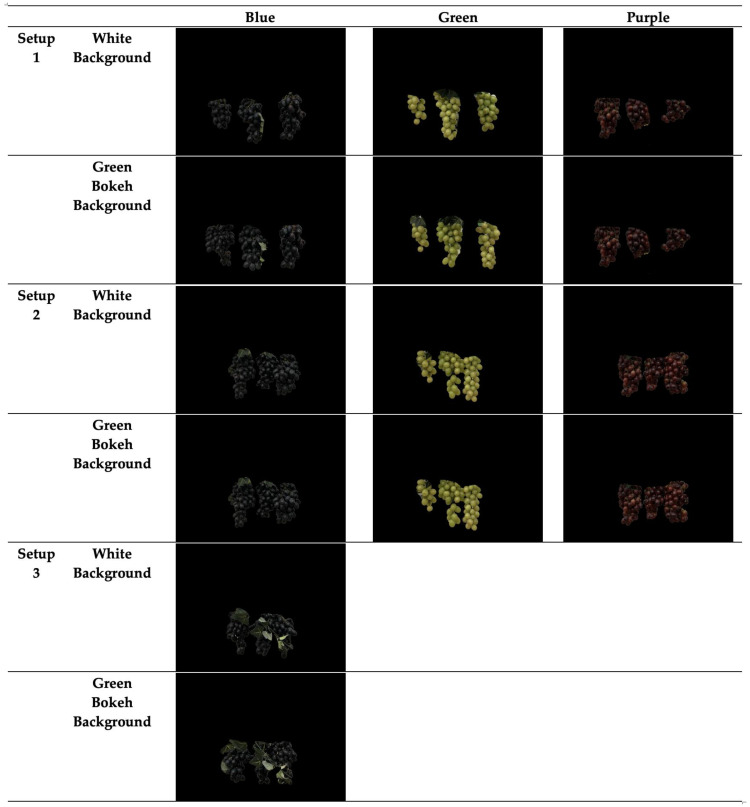
The segmentation results for blue, green, and purple grape clusters using GCNet.

The performance of the segmentation model was evaluated across different grape colors—blue, green, and purple—and the results are summarized in Table 5 and visualized in Figure 5. The IoU and F1 score metrics reveal how well the model performed for each color, while the qualitative results illustrate the accuracy of the segmentation model for different grape colors.

The model performed best on blue grapes, achieving an IoU of 0.93 and an F1 score of 0.97. These high values reflect the model’s ability to produce accurate segmentation masks that closely match the ground truth. The high contrast between the dark blue grapes and the background played a significant role in this result, allowing the model to easily distinguish the grapes from their surroundings. The uniformity in the color of the blue grapes further contributed to this success, as the model had a consistent feature to learn from, resulting in minimal errors during segmentation. In contrast, the model struggled the most with green grapes, which achieved the lowest IoU (0.82) and F1 score (0.92). The reduced segmentation accuracy for green grapes is likely due to the difficulty in distinguishing them from the surrounding green foliage and the green bokeh background. The similarity in color between the grapes and their environment created confusion for the model, leading to less precise segmentation. This outcome is expected when there is little color contrast between the object and the background, making it harder for the model to correctly identify the boundaries of the grape clusters. For purple grapes, the model achieved intermediate results, with an IoU of 0.87 and an F1 score of 0.95.

Although the contrast between the purple grapes and the background was better than for green grapes, the model faced some challenges due to internal variations in the shades of purple within the grape clusters. These subtle differences in color within the clusters likely caused slight inaccuracies in segmentation, particularly along the boundaries, but the overall performance remained solid due to the relatively good contrast with the background.

The qualitative results from the color experiment (Figure 5) demonstrate the segmentation model’s varied performance across different grape colors—blue, green, and purple—on two background types (white and green bokeh) and under different experimental setups. 

Blue grapes yielded the most accurate segmentation across both setups and backgrounds. The dark color and high contrast with both white and green backgrounds allowed the model to consistently capture well-defined cluster boundaries, resulting in minimal segmentation errors. The model effectively isolates each blue grape cluster without significant portions being missed or background noise being included, aligning with the highest IoU and F1 score observed for this color.

For green grapes, the segmentation is notably more challenging, especially with the green bokeh background, where the similarity in color between the grapes and the background introduces confusion. This similarity often causes the model to over-segment, inaccurately including parts of the background as grape clusters, particularly in Setup 2. On the white background, green grapes show a slight improvement in segmentation accuracy; however, occasional boundary inaccuracies remain, suggesting that contrast enhancement may be beneficial. The lower IoU and F1 score for green grapes reflect these difficulties, reinforcing that additional preprocessing or color distinction methods may be needed when green grapes are in natural settings.

Purple grapes present an intermediate case between blue and green grapes. The segmentation is generally accurate on the white background, with well-delineated grape clusters and only minor edge inaccuracies. However, on the green bokeh background, there are some minor errors due to background blending, particularly along the edges of the clusters. The internal variations in purple shades within each grape cluster likely contribute to these slight inaccuracies. Nevertheless, the purple grape segmentation remains reasonably accurate, as reflected by moderate IoU and F1 scores.

The qualitative analysis confirms that color contrast between grape clusters and their background significantly impacts segmentation accuracy. Blue grapes perform best due to high color contrast, while green grapes, with their lower contrast against natural backgrounds, pose the greatest challenge. Purple grapes fall in between, as internal color variations affect precision. These findings suggest that enhancing color contrast through preprocessing could further improve segmentation performance in real-world applications.

Overall, the Color Experiment demonstrates that the model’s segmentation accuracy is heavily influenced by the contrast between the grape clusters and the background. Blue grapes, with their strong contrast and uniform color, were segmented most accurately, while green grapes presented the greatest challenge due to their color similarity with the foliage and background. This is as expected, according to Mohimont et al. [47]. Purple grapes fell in between, as the model contended with internal color variations, but the overall contrast with the background was sufficient to achieve reasonably accurate segmentation. These findings suggest that in real-world applications, additional techniques, such as preprocessing to enhance contrast or using more advanced segmentation models, may be required when dealing with challenging cases like green grapes.

## 7. Discussion

This section discusses a comparative analysis of our results with prior studies in grape counting and segmentation, the key findings from the experimental results, highlighting the strengths and limitations of GCNet’s performance across different conditions, as well as its implications for real-world applications in grape yield estimation.

### 7.1. A Comparative Study with Prior Studies

The effectiveness of GCNet was evaluated against the backdrop of existing methods in grape segmentation and yield estimation, providing valuable context to assess its performance. Studies such as Zabawa et al. [6] and Santos et al. [11] applied deep learning techniques for grape segmentation, achieving an F1 score of 0.89. Zabawa et al. employed a U-Net variant specifically optimized for grape cluster segmentation, while Santos et al. used a similar U-Net-based framework with additional modifications to improve robustness under varying vineyard conditions. Similarly, Sozzi et al. [4] utilized traditional image processing approaches, which reported a lower F1 score of 0.77, reflecting the limitations of non-deep learning methods in handling complex vineyard imagery. On the other hand, Marani et al. [37] and Peng et al. [39] achieved an IoU of up to 88%, leveraging convolutional neural networks (CNNs) for grape detection and segmentation. These studies employed vineyard-specific datasets and optimized CNN architectures tailored to their respective conditions. These works underline the progress made in the field but also highlight the need for further advancements in accuracy, particularly under challenging conditions such as occlusions and low-resolution imagery.

In contrast, GCNet demonstrated significant improvements, achieving an IoU of up to 0.93 and an F1 score of up to 0.97 as demonstrated in Table 6. This performance can be attributed to the integration of a U-Net-based segmentation model and a correction factor to account for occluded grapes. The segmentation component effectively isolates grape clusters, even in images with varying resolutions and colors, while the correction factor enhances yield estimation by compensating for hidden grapes. These results position GCNet as a robust framework for grape segmentation, outperforming existing methods in accuracy and reliability. The comparison is based on results reported in prior studies. Each method was evaluated using its own dataset and experimental setup, as no common benchmark dataset for grape segmentation currently exists. While this limits direct comparability, we included these comparisons to provide context for GCNet’s performance relative to existing approaches.

### 7.2. Impact of Segmentation on Model Performance

The Ablation Study demonstrated the critical role of segmentation in improving GCNet’s performance for grape yield estimation. Across both the Resolution and Color Experiments, segmentation consistently enhanced the model’s accuracy, as evidenced by improvements in both R^2^ and MAE. The most notable gains were observed for low-resolution images and green grapes, where segmentation helped the model overcome challenges related to limited image detail and color similarity with the background. In the Resolution Experiment, the addition of segmentation led to a significant improvement in performance, particularly for low-resolution images. The R^2^ value for low-resolution images increased from 0.85 to 0.88, while the MAE decreased from 29 to 26. These results suggest that segmentation is essential when image resolution is insufficient to capture the fine details of the grape clusters. Higher resolution images benefit less dramatically from segmentation, as they inherently provide more pixel information for the model to work with. This finding aligns with the hypothesis that segmentation is most beneficial when the quality of the raw input data is lower. In the Color Experiment, segmentation provided the greatest benefit for green grapes, where the model saw a 0.3-point increase in R^2^ and a reduction in MAE from 33 to 29. The color similarity between green grapes and the background made it difficult for the model to differentiate between the two without segmentation. By focusing on isolating the grape clusters, segmentation mitigated the issue of background noise, leading to more accurate yield estimates. For blue and purple grapes, the improvements were smaller but still notable, reflecting the fact that segmentation helps to refine the model’s predictions even when color contrast is relatively high.

To evaluate the statistical significance of the observed 10% reduction in MAE with the inclusion of stage 3 in GCNet, a paired *t*-test was conducted. The paired *t*-test compares the MAE values obtained from experiments with and without stage 3 under identical conditions. This test was chosen to determine whether the reduction in MAE is statistically significant or attributable to random variation. The analysis was based on 30 paired samples of MAE values, obtained from repeated evaluations across different datasets and experimental setups. The assumptions of the paired *t*-test, including normality of the differences in MAE values, were validated prior to performing the test. A significance threshold of *p* < 0.05 was used to assess the results.

The results confirmed that the 10% reduction in MAE observed with the inclusion of stage 3 in GCNet is statistically significant. The paired *t*-test yielded a *p*-value of *p* = 0.012, *p* = 0.012, indicating that the improvement in MAE is unlikely to be due to random chance. The average MAE without stage 3 was 29, while the average MAE with stage 3 was reduced to 26, demonstrating the consistent impact of the additional regression step. This analysis was conducted under the same experimental setup to ensure consistency and validity.

These findings underscore the importance of stage 3 in enhancing GCNet’s performance for grape yield estimation, validating its contribution to the framework’s overall effectiveness. By leveraging features extracted during segmentation, the regression stage improves the model’s ability to account for occluded and overlapping grape clusters. Together, these results highlight the critical roles of segmentation and subsequent regression in achieving accurate grape yield estimation.

### 7.3. Influence of Image Resolution on Segmentation Accuracy

The Segmentation Study revealed a strong correlation between image resolution and segmentation performance. As demonstrated in the Resolution Experiment, segmentation accuracy, as measured by IoU and F1 score, improved consistently with increasing image resolution. At low resolution, the model achieved an IoU of 0.83 and an F1 score of 0.93, while at high resolution, these metrics improved to 0.90 and 0.96, respectively. This result highlights the importance of image resolution for tasks requiring precise segmentation, such as grape yield estimation. High-resolution images provide the model with more detailed information about the shape, texture, and boundaries of the grape clusters, enabling more accurate segmentation. However, the fact that the model still performed reasonably well on low-resolution images (with an IoU of 0.83) suggests that GCNet is robust enough to handle lower-quality inputs, although with some degradation in accuracy. From a practical standpoint, the trade-off between image resolution and computational efficiency must be considered. While higher resolution images yield better segmentation results, they also require more computational resources, both in terms of memory and processing power. Therefore, for real-world applications, it is important to balance the need for high segmentation accuracy with the cost of increased computational load. In contexts where computational resources are limited, medium-resolution images may offer an acceptable compromise between performance and efficiency. The framework operates efficiently on standard GPUs, processing images in just a few minutes without the need for supercomputer-level resources. This ensures accessibility and practicality for vineyard yield estimation tasks. In contexts where computational resources are constrained, medium-resolution images can provide an acceptable compromise between segmentation performance and efficiency.

### 7.4. Influence of Grape Color on Segmentation Performance

The Color Experiment in the Segmentation Study further illustrated how the model’s ability to segment grape clusters is affected by the color contrast between the grapes and their background. The model performed best on blue grapes, with an IoU of 0.93 and an F1 score of 0.97, owing to the strong contrast between the dark blue grapes and the background. In contrast, green grapes presented the greatest challenge, with the model achieving only an IoU of 0.82 and an F1 score of 0.92. This decline in performance can be attributed to the color similarity between the green grapes, the surrounding foliage, and the green bokeh background. These findings suggest that segmentation models such as GCNet are more effective when the object of interest has a distinct visual contrast from its surroundings. For blue grapes, the model was able to clearly distinguish the clusters from the background, resulting in highly accurate segmentation. However, for green grapes, the model often included parts of the background in the segmentation mask due to the visual similarity between the grapes and the leaves. The purple grapes presented an intermediate case, with an IoU of 0.87 and an F1 score of 0.95, reflecting the fact that while there was sufficient contrast, internal variations in the shade of purple within the clusters made segmentation more challenging. Blue grapes provide higher contrast against natural backgrounds, making segmentation easier. In contrast, green grapes blend with foliage, complicating segmentation.

### 7.5. Future Prospects for Grape Counting

Despite its strong performance, GCNet is not without limitations. The model’s vulnerability to densely packed clusters and overlapping berries remains a challenge, as the correction factor’s accuracy diminishes in scenarios with extreme occlusion. Additionally, the framework’s reliance on controlled indoor datasets limits its generalizability to outdoor vineyard conditions, where lighting and background variability introduce additional complexities. While the use of medium-resolution images addresses computational constraints, further optimization is needed to enable real-time scalability.

Future work will focus on addressing these limitations by expanding the dataset to include outdoor imagery and incorporating multi-angle imaging to reduce the impact of occlusions. Techniques such as 3D imaging, multi-scale context information extraction [73], multi-scale feature fusion and global attention mechanisms [74], or multi-spectral analysis could further enhance GCNet’s robustness across diverse conditions. Additionally, exploring lightweight model architectures through knowledge distillation or pruning could reduce computational demands, making GCNet more accessible for large-scale vineyard applications.

## 8. Conclusions

This study introduced GCNet, a novel deep learning framework for grape yield estimation, integrating a U-Net-based segmentation model and a correction factor to addressed occlude grapes. The results highlight the critical role of segmentation in improving yield estimation accuracy, particularly for challenging scenarios such as low-resolution images or visually similar grape colors. Segmentation significantly enhances R^2^ and MAE metrics, with the greatest impact observed for low-resolution inputs and green grapes, while high-resolution images provided marginal but consistent benefits.

Practical challenges, such as image quality and environmental variability, remain key considerations for deploying GCNet in real-world vineyards. High-resolution images improve segmentation performance but come with increased computational demands, necessitating a balance between image quality and efficiency. Future research should focus on improving segmentation for green grapes through contrast enhancement, 3D imaging, multi-angle image capturing, or multi-spectral imaging and optimizing the model for low-resolution images to enhance scalability. By addressing these limitations, GCNet holds significant potential as a robust tool for automated yield estimation in diverse vineyard environments.

## Figures and Tables

**Table 2:** The results of the ablation study comparing the performance of GCNet across different image resolutions (Low, Medium, High), both with and without the segmentation module.

Resolution	Without Segmentation		With Segmentation	
	MAE	R^2^	MAE	R^2^
Low	29	0.85	26	0.88
Medium	22	0.89	21	0.91
High	20	0.91	19	0.92
Average	24	0.88	22	0.90

**Table 3:** The results of the color experiment, comparing the performance of GCNet across different grape colors (Blue, Green, and Purple) with and without segmentation.

Grape Color	Without Segmentation		With Segmentation	
	MAE	R^2^	MAE	R^2^
Blue	13	0.95	13	0.96
Green	33	0.82	29	0.85
Purple	21	0.91	20	0.91
Average	22	0.89	21	0.91

**Table 4:** The quantitative results for the segmentation experiment, showing the Intersection over Union (IoU) and F1 score values for low-, medium-, and high-resolution images.

Resolution	IoU	F-1 Score
Low	0.83	0.93
Medium	0.87	0.95
High	0.90	0.96
Average	0.87	0.95

**Table 5:** The segmentation performance across three grape colors: blue, green, and purple. The results are presented in terms of Intersection over Union (IoU) and F1 score.

Grape Color	IoU	F-1 Score
Blue	0.93	0.97
Green	0.82	0.92
Purple	0.87	0.95
Average	0.87	0.95

**Table 6:** Comparison of IoU and F1 scores achieved by GCNet with other grape segmentation methodologies.

Study	Approach	IoU	F1 Score
Zabawa et al. [6]	Deep learning segmentation		0.89
Santos et al. [11]	Deep learning segmentation		0.89
Sozzi et al. [4]	Traditional image processing		0.77
Marani et al. [37]	CNN-based segmentation	0.88	
Peng et al. [39]	CNN-based segmentation	0.88	
(Ours) GCNet	U-Net-based segmentation + correction factor	0.93	0.97

## Data Availability

The GrapeSet dataset utilized and created in this study is publicly available and accessible at the following link: https://doi.org/10.5281/zenodo.14019981 (accessed on 22 January 2025). The created deep learning framework, GCNet, is publicly available and accessible at the following link: https://github.com/rubiquinones/GCNet (accessed on 22 January 2025).
